# The Treatment of Labour Complicated by Fibroid Tumours of the Uterus

**Published:** 1908-07-11

**Authors:** 


### Obstetrics.

### THE TREATMENT OF LABOUR COMPLICATED BY FIBROID TUMOURS OF THE UTERUS.

Fibroid tumours of the uterus present one of the most serious complications of labour in certain cases, and require prompt and deliberate treatment to avert fatal results. The effect produced by a fibroid on the course of labour depends very much on the site of the tumour and upon its size and consistence. The worst tumours in this respect are those which originate in the cervix. Such tumours are liable to grow into the connective tissue in front between the uterus and the bladder, or behind between the uterus and the rectum, and in both instances may remain beneath the level of the peritoneal reflections. The result is that the tumours are fixed down in the pelvis by the connective tissue and peritoneum, and cannot rise up during the enlargement of the uterus. It is clear, therefore, that such tumours will more or less fill the pelvis, according to their size, and may prove an insuperable bar to delivery at full term *per via naturales*. The same result may be produced by a pedunculated subperitoneal fibroid growing from the back of the uterus, which distends Douglas' pouch and is pressed down into the pelvis by the enlarging uterus and the presenting part.

In the case of fibroids in the uterine wall above the pelvic brim the effect upon labour is often nil, but there are cases on record in which the tumour projected so much into the uterine cavity that it moulded itself against some part of the fœtus, such as the groove between the occiput and back, so that the fœtus could not be forced past the obstruction. In other cases the presence of fibroids in the uterine wall has interfered with the normal uterine contractions, and even rupture of the uterus has been known to occur in their immediate vicinity. If a fibroid does not interfere with the expulsion of the fœtus, as a rule it does not affect the course of the third stage of labour or the involution of the uterus. However, even in this case there is sometimes a tendency to post-partum hæmorrhage, the fibroid being supposed to interfere with the normal contraction and retraction. This explanation is, however, quite doubtful, the probability being that the third stage of labour has not been properly conducted in cases where hæmorrhage has occurred, or that the uterus has been precipitately emptied by forceps when in a condition of secondary uterine inertia; the latter proceeding is invariably followed by bleeding, whether there is a fibroid in the uterus or not.

When a fibroid with full-time pregnancy is diagnosed, the question naturally arises, Can the tumour be pushed up out of the way of the presenting part or not? Clearly, if the tumour is of the kind first mentioned, it cannot be pushed up. If, however, it is pedunculated and only hanging down in front of the presenting part, there should be a possibility of pushing it up. This will be the more feasible the earlier in labour it is diagnosed. Such tumours have even been known to go up spontaneously after labour has been in progress some time. In some instances the character of the tumour may have been noticed at an examination in the early months of pregnancy, and then its treatment will be correspondingly easy. Since it may be sometimes possible to push up a fibroid above the presenting part, an attempt may be cautiously made under an anæsthetic; but it must not be forgotten that such a proceeding is not devoid of danger, as there is a risk of rupture of the uterus or of tearing some part of the attachment of the tumour to the uterus with consequent peritoneal hæmorrhage. Attempts at reposition should certainly be gentle and not prolonged. In a few cases there may be just enough room to extract the fœtus past the tumour, but this is fraught with great danger on account of the risk of rupturing the uterus or bruising the tumour, with subsequent gangrene or infection or laceration of its attachments. Injury to the tumour or its capsule must be the history of those cases in which fibroids have been spontaneously expelled from the uterus after delivery, nearly always with more or less serious sepsis. In cases where it is impossible to move the tumour or to deliver on account of filling of the pelvis, the only hope of safety lies in opening the abdomen as early in labour as possible, or, better still, just before labour begins. In such an operation a careful examination of the tumour and its attachment must be made to decide whether it can be removed first, labour being left to nature, or whether the uterus should be first emptied by Cæsarean section and then the tumour be left alone or dealt with at once. In general the second method will be found to be the safer. Myomectomy at all times is an operation which has been attended with a higher mortality than hysterectomy, and during pregnancy there are additional dangers from hæmorrhage and possible sepsis. It is far easier to perform hysterectomy during labour than to enucleate a fibroid from the uterus if delivery is to be then left to nature. Although some cervical fibroids look as if they might be enucleated from the vagina, this operation during the latter end of pregnancy would almost certainly be followed by severe hæmorrhage very difficult to control, and would further leave a large cavity which might readily be bruised and lacerated by the delivery of the fœtus. On the whole it is best to perform Cæsarean section, for this is the best chance of getting a living child; and then either to close the wound as in a conservative operation, leaving the fibroid alone, or at once to remove the uterus and fibroid together by a supra-vaginal amputation.

Occasionally it may be feasible to enucleate the tumour, but this will only be when its attachments to the uterus are narrow and when bleeding can easily be controlled. Hysterectomy just before or during labour is a very easy operation, and if no vaginal examinations have been made or the uterus infected the results are very favourable. If the uterus and tumour have been left, there is a very real danger of necrobiosis occurring, which will necessitate another laparotomy at no very distant date.

